# Catheter-Related *Acremonium kiliense* Fungemia in a Patient with Ulcerative Colitis under Treatment with Infliximab

**DOI:** 10.1155/2011/710740

**Published:** 2011-06-30

**Authors:** Fernando A. Díaz-Couselo, Marcelo Zylberman

**Affiliations:** Instituto Alexander Fleming, Crámer 1180. Ciudad Autónoma de Buenos Aires, C1426ANZ, Argentina

## Abstract

*Acremonium* spp. are filamentous, cosmopolitan fungi commonly isolated from plant debris and soil. They are infrequent pathogens in humans. *Acremonium* fungemia has been reported in neutropenic patients associated with central venous catheters and in nonneutropenic patients receiving long-term total parenteral nutrition. TNF-**α** blockade is associated with fungal infections, but no *Acremonium* spp. infection had been reported up to the present. In this paper, we present a patient with ulcerative colitis who developed *Acremonium kiliense* fungemia associated with infliximab therapy while receiving total parenteral nutrition. The patient was successfully treated with voriconazole. *Acremonium* sp. infection must be suspected as another cause of fungal infection in patients under treatment with infliximab.

## 1. Introduction

Infliximab is a chimeric IgG1*κ* monoclonal antibody used for the treatment of bowel inflammatory diseases, rheumatoid arthritis, and psoriasis. Infliximab binds to the soluble and transmembrane forms of tumor necrosis factor-*α* (TNF-*α*) receptors, neutralizing the biologic activity of TNF-*α*. TNF-*α* blockade is associated with invasive fungal infections [1]. 

In this paper, we report a patient with ulcerative colitis (UC) who developed *Acremonium kiliense* catheter-related fungemia associated with infliximab therapy while receiving total parenteral nutrition.

## 2. Case Presentation

A 30-year-old male patient was admitted because of diarrhea. He had a history of 14-month UC treated with mesalamine, glucocorticoids, cyclosporine, and 6-mercaptopurine (6-MP). When he was admitted, he was only on prednisone and 6-MP. Pancolitis was revealed by total colonoscopy. Despite glucocorticoid and 6-MP dose escalation, diarrhea increased. Total parenteral nutrition was started. Fever developed. Blood and urine cultures were negative. *Clostridium difficile* toxins (stool) and early cytomegalovirus antigen (stool and blood) were negative. Fluconazole was started upon evidence of *Candida *sp. growth in stool culture. The central venous catheter (CVC) was removed. The CVC tip culture was negative, and a new CVC was inserted. CT scan did not reveal any pulmonary infiltrates or intraabdominal abscess. Fever was assumed to originate from UC activity. Infliximab therapy (5 mg/kg) was initiated. The patient received two doses of infliximab (days 0 and 14). Diarrhea and fever continued. A total colonoscopy was performed 24 days after the first dose of infliximab. No improvement was recorded.

 Peripheral vein and CVC blood samples were cultured simultaneously. *Acremonium kiliense* was isolated in CVC blood samples more than 2 hours earlier than isolation from peripheral blood cultures. Catheter related fungemia was diagnosed. 

The CVC was removed, and intravenous voriconazole (6 mg/kg bid on the first day and then 4 mg/kg bid) was initiated. Blood cultures after 7 days of therapy were negative. A total colectomy, an ileostomy, and a mucous fistula were performed. The patient was discharged with oral prednisone and rectal budesonide. Oral voriconazole treatment was continued until glucocorticoids ended.

## 3. Discussion

*Acremonium* spp. are filamentous, cosmopolitan fungi typically isolated from plant debris and soil. However, they are infrequent pathogens in humans. In normal hosts, *Acremonium* sp. is the causative agent of eumycotic white grain mycetoma, onychomycosis, keratitis, endophthalmitis, peritonitis, whereas in immunocompromised hosts, it has been reported to cause catheter related infections, pneumonia, endocarditis, meningitis, brain abscesses and osteomyelitis [2]. In 1991, Fincher et al. reviewed case reports including *Acremonium* infections and classified them as (1) allergic, (2) colonizing, (3) superficial/locally invasive, (4) ocular, (5) mycetoma, (6) invasive pulmonary, or (7) disseminated [3]. *Acremonium* infections in immunocompromised patients appear to be rare. In patients with hematological malignancies, *Acremonium* was isolated in only one of 391 cases of filamentous fungi infections over a ten-year term [4].

*Acremonium* fungemia associated with CVC has been reported in neutropenic patients and in nonneutropenic patients receiving long-term total parenteral nutrition [5–8].

We have not found any *Acremonium* fungemia reports in association with infliximab, but fungal infections with TNF-*α* blockers have been well described. Tsiodras et al. reviewed 226 mycoses associated with infliximab, most of which were caused by *Histoplasma* sp. (*n*: 72), *Candida* sp. (*n*: 54), *Aspergillus* sp. (*n*: 48), *Coccidioides* sp. (*n*: 27), and *Cryptococcus* sp. (*n*: 17) [1]. None of them had been caused by *Acremonium* sp [1]. In the patient reported in this paper, *A. kiliense* was isolated 24 days after the first dose of infliximab. Mycoses associated with infliximab usually appear at a median of 55 days after initiation of therapy, but the interquartile range is between 15 and 140 days [1].

Successful treatment of *Acremonium* fungemia with voriconazole was reported by Mattei et al. [9].

Recommendations have been published to reduce the infection risk with the use of TNF-*α* blockers [10]. Recently, a European Consensus has alerted about the low-frequency but high mortality rate of patients with inflammatory bowel diseases who are under immunomodulatory treatments and develop fungal infections. This Consensus has advised that when a fungal or parasitic infection occurs, TNF-*α* blockers must be withdrawn. These agents may be reintroduced in conjunction with secondary chemoprophylaxis [11].

This seems to be the first case of *A. kiliense* fungemia in a UC patient receiving infliximab. *Acremonium* sp. infection must be suspected as another cause of fungal infection in patients under infliximab treatment.
